# Cost savings of outpatient parenteral antimicrobial therapy using a digital infusion pump

**DOI:** 10.1093/jacamr/dlag052

**Published:** 2026-05-06

**Authors:** Rita Helleren, Marianne Jacobsen, Theis Theisen, Carl Erik Moe, Anne Opsal, Rune Trønnes, Vegard Skogen, Frode Gallefoss

**Affiliations:** Department of Internal Medicine, Sørlandet Hospital, Kristiansand, Norway; Department of Internal Medicine, Sørlandet Hospital, Kristiansand, Norway; Department of Economics and Finance, School of Business and Law, University of Agder, Kristiansand, Norway; Department of Information Systems, University of Agder, Kristiansand, Norway; Department of Health and Nursing Science, University of Agder, Kristiansand, Norway; Department of Patient Administration, Sørlandet Hospital, Kristiansand, Norway; Department of Clinical Medicine, Faculty of Health Sciences, UiT, The Arctic University of Norway, Tromsø, Norway; Department of Infectious Diseases, Division of Internal Medicine, University Hospital of North Norway, Tromsø, Norway; Department of Internal Medicine, Sørlandet Hospital, Kristiansand, Norway; Faculty of Medicine, University of Bergen, Bergen, Norway

## Abstract

**Background and objectives:**

Outpatient parenteral antimicrobial therapy (OPAT) enables IV antibiotic administration outside hospital settings and has been found to reduce costs and improve patient satisfaction. In Norway, OPAT has been less established due to the preference for narrow-spectrum antibiotics often requiring multiple daily doses. We undertook a cost evaluation of an OPAT programme using a continuous ambulatory delivery device (CADD), and comparison with costs of inpatient treatment for equivalent IV antibiotic courses.

**Materials and methods:**

A retrospective cost analysis was conducted on 170 consecutive patients treated with OPAT at Sørlandet Hospital, Norway (2016–2021). Eligible patients received multidose IV antibiotics at home using CADD pumps. OPAT costs included equipment and antibiotics, staff time, transportation and patient time, and were compared with estimated inpatient costs for the same treatment and duration. Sensitivity analyses tested robustness of the findings across various cost scenarios.

**Results:**

A total of 170 patients received OPAT, mostly with narrow-spectrum β-lactam antibiotics, with a mean duration of 16.4 days per patient (range 1–67 days), and an estimated saving of approximately 51 hospital bed days per month. The mean cost per OPAT treatment was €1603 versus €12 982 for inpatient care, i.e. representing 12% of inpatient costs. Sensitivity analysis confirmed OPAT savings between 72% and 89% across scenarios.

**Conclusions:**

These findings confirm that a CADD-based OPAT programme considerably reduces costs on a societal perspective in a publicly funded health system and contributes to reduced hospital bed occupancy. The ability to administer narrow-spectrum antibiotics supports antimicrobial stewardship.

## Introduction

Outpatient parenteral antimicrobial therapy (OPAT), defined as at least two doses of parenteral antibiotic treatment administered on different days without intervening hospitalization,^1^ has been used in clinical practice since the early 1970s.^2^ Cost reductions and increasing patient satisfaction have been key objectives from the start.^2^ OPAT has shown to be cost-effective and safe for several infections,^3^ to reduce bed days,^4,5^ reduce hospital-acquired infections^6^ and reduce environmental impact of the treatment.^7^

OPAT favours the use of antibiotics with a long half-life such as ceftriaxone, daptomycin and ertapenem.^1,8^ This differs from the Norwegian national treatment guidelines for antibiotic use in hospitals, which promote the use of narrow-spectrum β-lactam antimicrobials that often require multiple daily doses.^9^ Furthermore, in Norway the population is widely dispersed, often with long distances between the hospitals and patients’ homes. This impediment, combined with the limited availability of functional devices that support multidose regimens, has contributed to OPAT being less established in Norway. However, the introduction of digital devices has made it increasingly feasible to administer antibiotics requiring multiple daily doses through OPAT.^1^

In 2016 an OPAT programme was established at Sørlandet Hospital Kristiansand, Norway. The programme implemented a modern, digital, continuous ambulatory delivery device (CADD) for home treatment allowing narrow-spectrum antimicrobials. Previous reports have shown that this programme achieves high patient satisfaction^10^ and is both efficient and safe.^11^

The Norwegian healthcare system is predominantly public and tax financed. Evaluations of cost-consequences of OPAT from a societal perspective, using a modern digital infusion pump, have to our knowledge not previously been published. The present study aimed to investigate the potential cost savings of a CADD-based OPAT programme. The study was from a societal perspective, accounting for costs incurred by all stakeholders, including patients’ time costs, and comparing these with the estimated costs for patients remaining hospitalized for the entire treatment period.

## Materials and methods

### Ethics

The Regional Committee for Medical Research Ethics in South-East Norway evaluated the project and defined it as not a part of their mandate. The study was approved by the Norwegian Agency for Shared Services in Education and Research (SIKT) in Norway (ID 60290/EPA/LR), the hospital’s data protection officer and the research department.

### Patients and OPAT programme

The OPAT programme was based on the use of a CADD (CADD Solis VIP infusion pump; Smith Medical, UK) and could be initiated at discharge or from the outpatient clinic directly. Consecutive patients were recruited during clinical practice. Patients were eligible if they were to receive parenteral antibiotics at home at least twice daily and consented to OPAT. All included patients were assessed as suitable for OPAT by their treating physician, i.e. they had to be medically stable and with a plan for antibiotic treatment. Patients over 18 years of age (no upper limit) were considered for the study. The patient or their caregiver had to be able to manage the CADD and give notice if any difficulties occurred between visits. Treatments with antibiotic dosage once daily were not included, because these patients did not receive treatment using CADD.

A broad variety of antibiotics were accepted. Because of possible drug instability at room temperature over time, meropenem was not administered as OPAT in this study.^12,13^ All antimicrobials were prepared once daily. For cloxacillin, cephalosporins, piperacillin/tazobactam, clindamycin and vancomycin, one batch was prepared daily, with a durability of 24 h.^14,15^ For benzylpenicillin and ampicillin, two batches were needed to ensure safety and stability,^14,16^ and one batch was kept cold in the refrigerator for 12 h before the patient changed the batch themselves. No buffers were used during preparation.

An infectious disease specialist was consulted before each course of OPAT to ensure correct antibiotic, dosage and treatment length. Patients received antibiotics through three alternative venous routes, i.e. either a peripheral venous catheter (PVC), a peripherally inserted central catheter (PICC) or a midline catheter (MC), according to treatment length and venous access.

Patients with either a PICC or MC and with a treatment duration of more than 7 days had follow-up both at the hospital and with a community nurse. They met a community nurse 5 days a week, and a nurse at the hospital infusion centre the remaining 2 days a week. Patients with shorter courses of treatment (7 days or less) or peripheral venous access attended the hospital infusion centre daily. All patients had blood tests taken twice a week and met with their responsible hospital specialist twice a week. In addition, all patients had access to the hospital by phone 24 h per day, in case of questions or complications.

Data were collected and categorized retrospectively and analysed by means of SPSS^®^ (Statistical Package for the Social Sciences v 29.0.0.0). Demographic data, type of infection, antimicrobial drug and dosage, number of days treated as inpatient and with OPAT, venous access, readmissions, complications and patient satisfaction were collected. These data have been published elsewhere.^10,11^

### Analysis

The actual costs of OPAT treatment were compared with the estimated costs as if the same patient had remained hospitalized with the same treatment for the entire treatment period. The total costs were measured in Norwegian kroner (NOK) per 2023, but to improve readability for international readers all costs are in the sequel converted to euros (€), using the 2023-average currency rate of €0.0876 per NOK.^17^ The economic analysis was performed from a societal perspective, including costs related to patient time, equipment and transportation, in addition to CADD-related time costs for the medical staff at the hospital, and the community nurses following patients in the programme. A societal perspective was chosen both to compare our results with previous studies, and to satisfy the recommendations to include patient costs in different contexts.^18,19^

### OPAT costs

All OPAT patients were equipped with a CADD, which requires only minimal training to operate. At the start of the OPAT programme, there was a need for 15 CADDs in continuous use, with an average 8 year life range. Cleaning, service and patient training were estimated as 0.5 nursing hours per OPAT treatment. Each patient received a non-reusable carry-on shoulder bag for the CADD. Patients treated with benzylpenicillin and ampicillin were given a small cooler to keep the second batch of antibiotics cold between the hospital and the refrigerator at home. Batteries were changed every fourth day. Peripheral catheters were changed every second day. For PICCs and MCs the infusion set was changed twice a week at the hospital infusion centre, of which one change coincided with full care of the venous access.

The hospital pharmacy (Sykehusapoteket HF) provided the actual antibiotic costs. Laboratory costs for OPAT patients were calculated using the price list provided by the Unit for Laboratory Medicine (R. Trønnes, personal communication). Nursing costs were based on average nurse wages according to statistics provided by the Norwegian nurses’ organization, with different wages for nurses working in a hospital and nurses working in community service.^20^ The average salary for an attending physician, provided by the Norwegian Medical Association (J. Hellwege, personal communication), was used for the calculation of doctors’ costs. All salary costs included social costs.

For those with follow-up in community health care 5 days a week and at the infusion centre twice a week, every nurse’s visit was assumed to last 1 h. When daily attendance at the infusion centre was required, five weekly nurse visits were assumed to last 0.5 h each, whereas two visits per week were assumed to last 1 h due to care of the venous access. All patients met their physician twice a week, each visit estimated at 0.5 physician hours.

Transportation costs were calculated assuming that all patients drove their own car. The official Norwegian state rates per kilometre for 2023 were used. Average hourly time costs for patients were calculated in accordance with the principles outlined by Flügel *et al*.^21^ For the OPAT group, only the time spent with the community nurse/at the hospital infusion centre, the time for doctor’s visits, and the time used to drive to/from the hospital, were included as time costs.

### Inpatient costs

Inpatient expenses per ‘bed day’ were based on data from Sørlandet Hospital’s accounting and finance department, with costs differing between the wards to which the patients were admitted. Cost per bed day included nursing costs, medication, maintenance costs and costs for supporting staff. Costs for laboratory and radiology analyses and doctors’ visits/work are not a part of cost per bed day and were therefore added separately.

Calculated costs for inpatient treatment instead of OPAT were based on the assumption of a continuous stay at the discharging ward for the same condition and length of treatment. Considering that patients were stable and had a defined diagnosis, the costs for laboratory and radiology investigations for inpatients were assumed to amount to 25% of the average cost for laboratory/radiology per bed day in the medical ward in 2023. Doctor visits were assumed to last 0.5 h per day. Inpatient time costs have not been added.

### Cost-consequence analysis

The aim was to compare the actual OPAT costs for defined infections requiring IV antibiotics with the estimated inpatient costs for equivalent antibiotic treatments over the same duration.^22^ Thus, OPAT costs are based on the actual treatment whereas inpatient costs have been estimated according to best available cost estimates. Completed IV antibiotic treatment was the most important clinical outcome and was defined as OPAT given as planned without complications directly associated with OPAT. Other clinical outcomes from the same programme, including readmissions, have previously been reported.^10,11^

All economic calculations were carried out exactly, but when presented, a rounding of the numbers to the closest euro is shown to increase readability.

A sensitivity analysis was performed to investigate the robustness of the results. For both groups higher and lower costs were calculated and later compared. In both the inpatient group and the OPAT group different scenarios were considered, with further details provided in the Results section.

## Results

### Patient demographics

The first 221 patients who received treatment with the OPAT programme at Sørlandet Hospital were eligible and invited to participate in this study. Patients with two or more OPAT treatments in the period were only invited once. Of the 221 patients invited, 51 were excluded (37 due to no written consent and 14 due to incorrect registration). Hence, a total of 170 consecutive patients treated between November 2016 and May 2021 consented and were included in the study (Table 1).

**Table 1.** Patient characteristics, diagnosis, microbiology and antibiotics

Characteristic		
Sex and age		
Male, *n* (%)	108 (64)	
Age, mean (SD), y	62 (16)	Range 19–93
Employment, *n* (%)		
Full-time	52 (31)	
Part-time	12 (7)	
Student	2 (1)	
Retired	75 (44)	
Other/missing	29 (17)	
Hospital stay		
Days hospitalized before OPAT, median, (25th/75th percentiles), mean (SD)	8 (4/15) 8 (11)	Range 0–71 days
ICU/intermediate care before OPAT, *n* (%)	17 (10)	
Intermediate care before OPAT, *n* (%)	13 (8)	
Tertiary hospital before OPAT^a^, *n* (%)	14 (8)	
Surgery before OPAT, *n* (%)	45 (26)	
Days treated with OPAT, median (25th/75th percentiles), mean (SD)	13 (8/23) 16.4 (12)	Range 1–67 days
Venous access, *n* (%)		
PICC line	139 (82)	
Midline	9 (5)	
PVC	20 (12)	
CVC	2 (1)	
Diagnosis, *n* (%)		
Endocarditis	28 (17)	
Bone and joint infections	53 (31)	
Postoperative infections	46 (27)	
Others	43 (25)	
Microbiology, *n* (%)		
Streptococci	29 (17)	
Enterococci	10 (6)	
Staphylococci	55 (32)	
Gram-negative rods	11 (6)	
Polymicrobial flora	8 (5)	
Others	22 (13)	
No microbe found	35 (21)	
Antibiotics, *n* (%)		
Benzylpenicillin	61 (36)	
Ampicillin	13 (8)	
Cloxacillin	63 (37)	
Second- and third-generation cephalosporins and piperacillin/tazobactam	22 (13)	
Others	11 (6)	

CVC, central venous catheter; PICC, peripherally inserted central catheter; PVC, peripheral venous catheter.

Numbers with decimals are rounded to nearest whole number, to increase readability. Percentages thus may not add up to 100 exactly.

^a^Tertiary hospital: university hospital (i.e. Oslo University Hospital).

**Figure 1.**
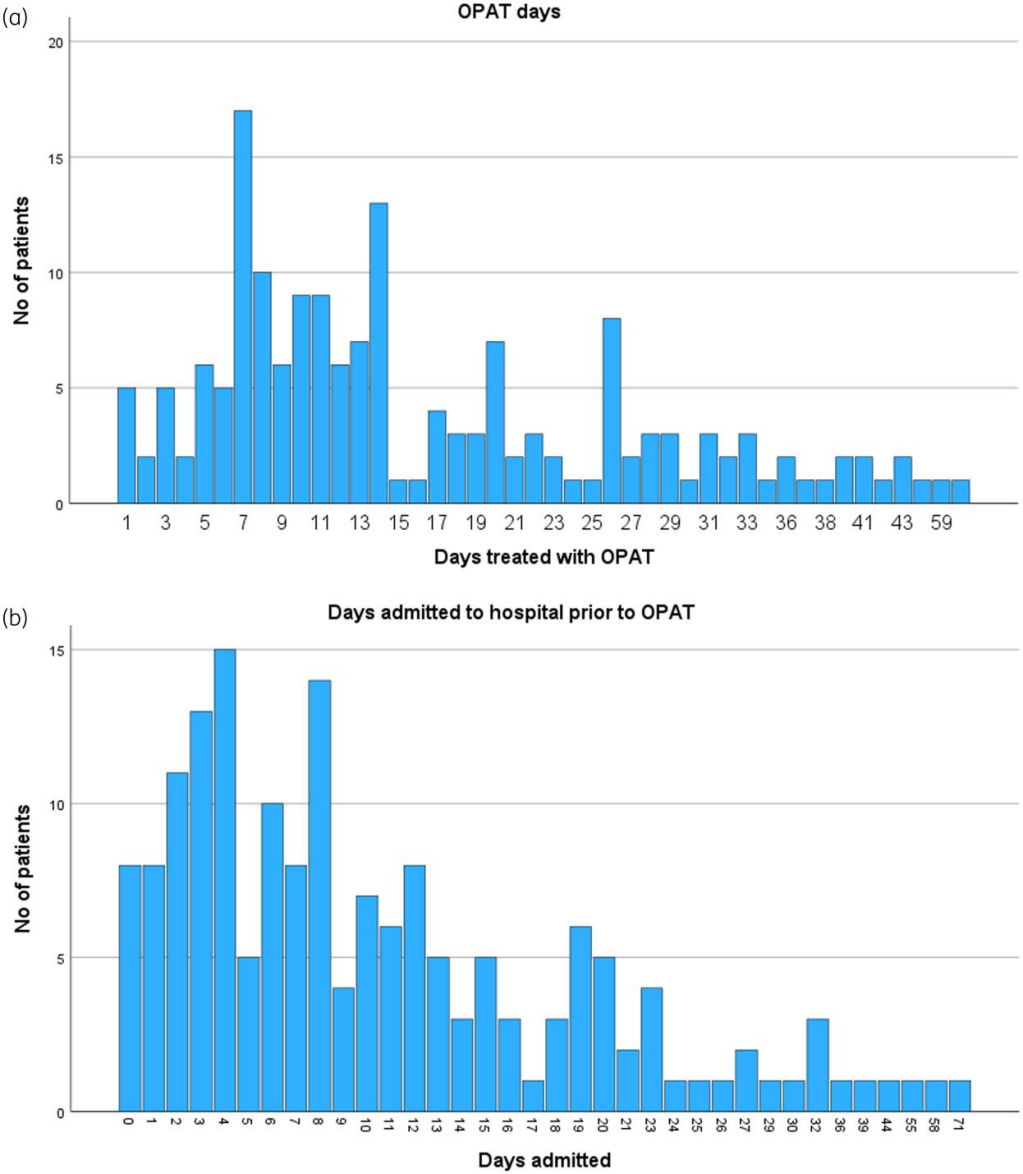
Distribution of OPAT days and days admitted to hospital prior to OPAT in our cohort.

**Figure 2.**
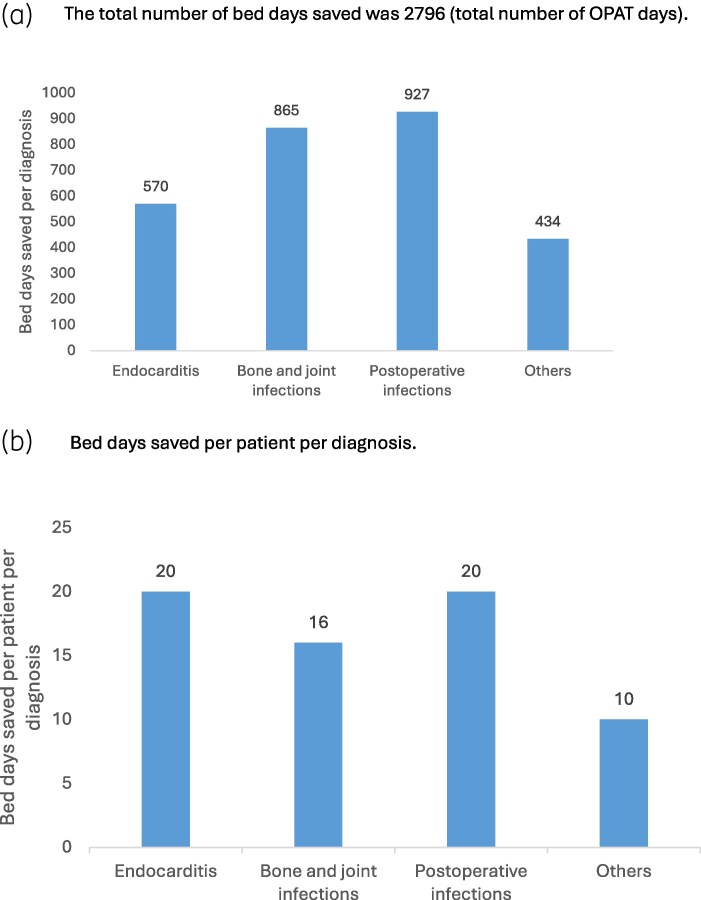
Number of bed days saved per diagnosis and per patient per diagnosis. (a) The total number of bed days saved was 2796 (total number of OPAT days). (b) Bed days saved per patient per diagnosis: 28 patients were treated for endocarditis, 53 patients for bone and joint infections, 46 for postoperative infections and 43 for other infections. All values are rounded to the nearest whole number.

One-fifth of the patients did not have a proven microbe. Staphylococci were the most frequently treated microorganism (33.3%) among patients in the study. Narrow-spectrum β-lactam antibiotics were administered to 80% of the patients (Table 1). A total of 162 patients were admitted to hospital prior to OPAT, whereas 8 patients started OPAT directly without being hospitalized. A total of 78 patients (46%) were discharged from the infectious diseases ward. The median number of inpatient days prior to OPAT was eight. The mean and median numbers of OPAT days per patient were 16.4 and 13, respectively. Distribution of OPAT days and days admitted before OPAT are shown in Figure 1. The total number of OPAT days was 2796, with bone/joint infections and postoperative infections accounting for 865 days and 927 days, respectively. Both endocarditis and postoperative infections accounted for more bed days saved per patient (Figure 2).

Readmissions from OPAT to inpatient treatment amounted to a total of 15 patients, with 9 due to possible worsening of the infection (two staphylococcal infections, two streptococcal infections, one *Aggregatibacter* infection, one polymicrobial infection and three patients where no microbe was found). The total number of readmission days was 48; none of these were directly related to OPAT. Further clinical details have been previously published.^10,11^

### Costs

**Figure 3.**
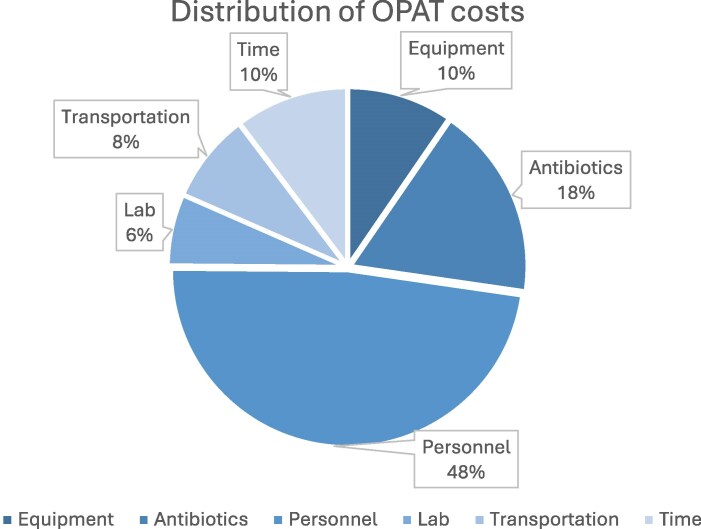
Distribution of OPAT costs. Personnel: Salary expenses for the community nurse, hospital nurse and specialist visits. Equipment: All equipment costs including sets for venous access, bandages, syringes, infusion fluids, batteries, cooler, carry-on bag, costs for the CADD, and costs for cleaning and maintenance of the CADD. Time (patient time costs): Only time spent with a nurse/doctor and time for transportation to/from the hospital. Transportation: Costs for the patient and the community nurse to/from the hospital and/or the patient’s home. Lab: Laboratory tests conducted twice weekly, including vancomycin levels for patients treated with vancomycin. Antibiotics: Costs for dry matter.

Table 2 presents the distribution of costs for OPAT and the estimated costs for the alternative inpatient stay. The mean costs of OPAT versus inpatient treatment were €1603 and €12 982, respectively. Thus, the mean cost for a completed OPAT treatment in our cohort was just 12% of the inpatient costs. The mean direct costs were 11% of the mean inpatient costs. The mean cost saving per patient receiving OPAT compared with inpatient care in this study amounted to €11 379. The difference in costs is largely explained by the substantial difference between ward costs for inpatient treatment, and costs related to equipment, antibiotics, staff and transportation for OPAT treatment. Whereas ward costs were the largest cost item in the inpatient segment, personnel costs accounted for almost 50% of OPAT costs (Figure 3). As none of the readmissions were directly related to complications from OPAT, readmission costs were excluded from the base case cost calculations. Hospitalization costs incurred prior to the initiation of OPAT in this comparison were logically excluded from the base case analysis.

**Table 2.** Direct, indirect and total costs: euros per patient for OPAT compared with inpatient treatment

	OPAT *n* = 170		Inpatient *n* = 170		Treatment difference, mean
	Mean (SD)	Median (25th/75th percentile)	Mean (SD)	Median (25th/75th percentile)	
Cost items					
Ward costs^a^	0	0	11 661 (8764)	9399 (5105/15 622)	
Lab inpatient (48 euros/day)	0	0	796 (574)	629 (387/1113)	
Doctor inpatient (64 euros/h)	0	0	525 (378)	415 (256/734)	
Equipment costs^b^	153 (68)	131 (98/199)	0	0	
Antibiotic costs	286 (318)	147 (78/406)	0	0	
Lab costs (6 euros/day)^c^	104 (99)	74 (46/142)	0	0	
Doctor costs (64 euros/h)	149 (104)	117 (72/206)	0	0	
Nursing costs			0	0	
Community nurse (40 euros/h)	420 (383)	364 (0/648)			
Hospital nurse (38 euros/h)	196 (118)	171 (115/267)			
Transportation costs (0.4 euros/km)			0	0	
For community nurse	43 (77)	17 (0/45)			
To/from the hospital	86 (101)	45 (17/111)			
Total direct costs	1438 (1061)	1064 (628/2145)	12 982 (9689)	10 363 (5766/17 271)	11 545
Patient time costs inpatient	0	0	0	0	
Patient time costs OPAT (7 euros/h)	166 (124)	122 (76/248)	0	0	
Total indirect costs	166 (124)	122 (76/248)	0	0	
Total costs	1603 (1182)	1185 (719/2386)	12 982 (9689)	10 363 (5766/17 271)	11 379

^a^Ward costs vary between 362 euros per bed day and 783 euros per bed day, with the highest costs for admittance to the infectious diseases ward.

^b^Equipment costs per patient: venous access equipment including syringes and NaCl for flush (3.2 euros per treatment day), batteries (0.15 euros per treatment day), cooler (1.5 euros per bag), carry-on bag (52 euros per bag), infusion fluids (1 euro per treatment day), CADD costs (0.6 euros per treatment day, buying price 1735 euros), costs for maintenance and cleaning of CADD (19 euros per treatment).

^c^OPAT patients had lab tests taken twice a week (haemoglobin, leucocytes with differential counting, thrombocytes, erythrocyte sedimentation rate, potassium, sodium, C-reactive protein, creatinine; liver function tests and vancomycin levels if indicated).

### Sensitivity analysis

Table 3 summarizes the results from a simple sensitivity analysis. To test the robustness of our base case cost analysis, minimum OPAT and inpatient cost alternatives were compared with the reciprocal maximum costs. Thus, patients admitted to the ward with the lowest cost per bed day (i.e. a patient hotel) and who had laboratory tests carried out twice a week (same as for the OPAT group) were compared with the highest OPAT cost alternatives, and vice versa. OPAT costs for the minimum total cost savings alternative were 28% of the equivalent inpatient costs, with a mean cost saving per patient day of €288; thus, OPAT saved 72% of inpatient costs. By contrast, in the maximum total cost saving alternative, assuming admittance to the ward with highest bed day costs (infectious diseases department) and comparing this with the least expensive OPAT alternative (OPAT with daily attendance at the hospital infusion clinic), total OPAT costs were 11% of the equivalent inpatient costs, with a mean cost saving per patient day of €771. Thus, the sensitivity analysis confirmed that OPAT saves between 72% and 89% of in-hospital costs.

**Table 3.** Sensitivity analysis of costs showing mean total cost changes (euros) under varying assumptions

	Cost savings^a^ per patient day	Cost savings^a^ per patient
Alternatives	Euros/day (inpatient cost − OPAT cost)	Euros/day (inpatient cost − OPAT cost)
Base case saving	692 (789 − 97)	11 379 (12 982 − 1603)
Minimum total cost savings^b^	288 (400 − 112)	4732 (6579 − 1847)
Maximum total cost savings^c^	771 (864 − 93)	12 683 (14 203 − 1520)

^a^All cost savings are mean total cost savings.

^b^For the calculation of minimum total cost savings, all patients were assumed to be admitted to the ward with lowest costs (362 euros/bed day) and to perform lab/radiology as OPAT patients (lab tests twice weekly), and to compare this with OPAT costs including costs for readmission days [in total 48 readmission days, i.e. 0.28 (mean) readmission days per patient. All readmission days were calculated assuming admittance to the ward with highest bed day costs (783 euros/bed day)].

^c^For the calculation of maximum total cost savings, the calculations of costs assumed all patients to be admitted to the ward with highest costs (infectious diseases department, 783 euros/bed day) compared with costs as if all OPAT patients had follow-up only at the hospital infusion centre.

## Discussion

This study confirms the potential for cost reductions of OPAT from a societal perspective using a CADD for administration of mostly narrow-spectrum β-lactam antibiotics, and indicates that OPAT treatment with CADD accounts for merely 12% of total costs when compared with the estimated inpatient costs. A sensitivity analysis confirmed considerable robustness towards substantial changes in the main cost assumptions in our analysis.

A limitation of the study is that data were retrospectively collected. However, the information necessary for robust economic analyses was easily accessible. In our OPAT programme laboratory tests were carried out twice a week, although updated guidelines only recommend at least once a week.^1,3^ We nevertheless chose two weekly blood tests for safety reasons because our OPAT programme mainly administered narrow-spectrum antibiotics with which there is less experience. A reduction in blood test frequency will be considered in the future, and this should further reduce the costs of OPAT treatment.

Our results are consistent with those of a Canadian study where OPAT costs were 13% of inpatient costs in the hospital perspective,^23^ and a British cost-consequence analysis from 2018 which found that OPAT costs amounted to 15% of inpatient costs, compared with the patient having been admitted to an infectious diseases ward.^4^ None of these studies were done with the use of the CADD pump, and none of them included societal costs.

In the UK, home-based treatments have for years been an important measure to reduce healthcare costs, increase patient satisfaction and combat the rise in costly hospital-acquired infections.^24^ Previous studies have also shown that OPAT, given by a specialist nurse at home or administered by the patient, is cost-effective^25^ and that OPAT leads to cost reduction compared with hospital admissions for the same treatment.^23^ Cost savings have also been shown in self-administered OPAT by continuous infusion through elastomeric pumps in a county hospital in Texas (USA)^26^ and in a Spanish ‘hospital at home’ setting.^27^ Even so, these studies are not directly comparable to the Norwegian healthcare setting, because the funding and expected costs are different. Moreover, no previous studies have compared the costs related to a CADD-based OPAT programme versus inpatient costs for the same antibiotic treatment.

Our CADD-based OPAT study implies a saving of 2796 bed days for this group of patients, an average of 51 bed days per month. OPAT is estimated to have saved a mean of 16 bed days per treatment. As the bed occupation rate in our hospital was above 85% in the same period, it is likely that another patient would fill the bed that was made available due to OPAT. Hence, OPAT has most likely contributed to reduced need for corridor beds/bed expansions, and less overtime for staff. The reduction of bed days also most likely contributes to less crowded and hectic hospital wards, which also may reduce hospital-acquired infections and spread of MDR microbes.^28^ The introduction of CADD OPAT may thus indirectly contribute to even larger hospital savings than our numerical results show, as well as reduced morbidity and mortality. Although absolute cost estimates in our study are highly context-specific and depend on local healthcare systems and cost structures, relative cost ratios between OPAT and inpatient care are more likely to be transferable across settings. Besides ‘completed intravenous antibiotic treatment’ as an important clinical outcome, patients have previously reported high patient satisfaction^10^ and the programme has been reported to be both efficient and safe.^11^ Further, being able to live at home during treatment most likely will improve quality of life compared with being treated as inpatient. None of the patients experienced complications directly associated with OPAT.

In Norway, the use of OPAT and established OPAT programmes varies considerably between hospitals. Lack of feasible devices that can administer narrow-spectrum antibiotics may contribute to this variation. With the increasing focus on cost-effective treatment programmes, the use of OPAT in Norway is likely to increase. In our opinion, the possibility of narrow-spectrum regimens administered by a CADD is the preferred method to achieve increased use. We have chosen a CADD-based programme in our hospital. The purchase cost of the CADD pump was €1735 in 2023. Each pump has a range of life of at least 8 years, and in this programme, pump cost per treatment day was €0.6. Compared with elastomeric pumps the costs for CADD pumps are significantly lower. The elastomeric pumps are changed either with each dosage or once/twice daily for a continuous infusion. With a cost of €39 (M.Jacobsen, personal communication) per elastomeric pump, the CADD has a significantly lower cost per treatment. In addition, the CADD requires a minimum of patient training and delivers antibiotics at a set time. Volume is not a challenge with the CADD pump and despite multidose treatments, the antibiotics may be prepared once or twice daily, which also facilitates multidose narrow-spectrum antibiotics.

Antimicrobial resistance is an increasing challenge worldwide, and a threat to modern healthcare. The yearly costs associated with antimicrobial resistance in Europe are estimated to exceed €11.7 billion.^29^ An important strategy for preventing resistance is the use of narrow-spectrum antibiotics.^30^ OPAT has recently been recognized as a tool in antimicrobial stewardship.^31^ It is, however, important to use narrow-spectrum antibiotics if OPAT is to play a significant role in antibiotic stewardship programmes. The use of narrow-spectrum antibiotics in OPAT can help reduce the environmental burden and selection pressure that drive antimicrobial resistance.

This study was conducted before the implementation of newer treatment regimens, which now generally recommend shorter treatment durations and, where possible, an earlier transition from IV to oral therapy.^9,32–34^ It is paradoxical that some of the recommended oral regimens involving broad-spectrum antibiotics, like the POET trial,^33^ should be used cautiously to avoid misuse and prevent further antimicrobial resistance. Although IV narrow-spectrum antibiotics may have a more favourable profile for both the patient and the environment, they should still be considered as viable options for certain patients otherwise considered for the transition to oral broad-spectrum antibiotics. In our opinion, OPAT continues to play a vital role alongside complex outpatient antimicrobial therapy, especially as the increased use of oral antimicrobials is becoming a rapidly evolving clinical practice.

Previous publications from the present study have reported high patient satisfaction and safety.^10,11^ The present study concludes that there are significant and robust cost reductions entailed by replacing inpatient treatment with a CADD-based OPAT programme. This study confirms the possibility to give narrow-spectrum multidose antibiotics as OPAT, which again should lead to further attention to and investigations of the increased use of this course of treatment.
